# Motor fatigue and fatigability in early multiple sclerosis patients: an analysis of clinical, radiological and psychological underpinnings

**DOI:** 10.1007/s10072-026-08988-4

**Published:** 2026-03-27

**Authors:** Matteo Betti, C. Masciulli, I. Addazio, C. Ballerini, R. Bonacchi, A. Caporali, F. Chiappetta, E. De Meo, C. Fabbiani, F. Gerli, U. Kihlbom, C. Niccolai, G. Pasquini, L. Pastò, V. Penati, K. Scholin Bywall, Jennifer Viberg Johansson, E. Fainardi, S. Martin, E. Portaccio, MP. Amato

**Affiliations:** 1 Department of NEUROFARBA, University of Florence, Viale Gaetano Pieraccini, 6, Florence, 50139 Italy; 2 Vita-Salute San Raffaele University, Milan, Italy; 3 Queen Square Multiple Sclerosis Centre, Department of Neuroinflammation, University College London (UCL) Queen Square Institute of Neurology, NMR Research Unit, London, UK; 4 Neuroradiology Unit, Department of Experimental and Clinical Biomedical Sciences Mario Serio, University of Florence, Florence, Italy; 5 IRCCS Don Carlo Gnocchi Foundation, Florence, Italy; 6 Center for research and bioethics, Uppsala University, Uppsala, Sweden; 7 Karolinska Institutet, Stokholm, Sweden; 8 Malardalens University, Västerås, Sweden

**Keywords:** Multiple Sclerosis, Fatigue, Motor Fatigability, 6MWT, Multidimensional assessment

## Abstract

**Background:**

While fatigue is highly reported in newly diagnosed people with Multiple Sclerosis (pwMS) and motor fatigability is reported in about 20% of non-disabled patients, relationship among them in early MS has been less studied.

**Objective:**

To evaluate correlations between fatigue and motor fatigability in early pwMS, and their clinical, radiological, psychological underpinnings.

**Methods:**

Relapsing pwMS aged 18-65 years, Expanded Disability Status Scale (EDSS) score <2.0, disease duration <5 years were recruited. PwMS underwent clinical, cognitive, radiological assessment. They performed a 6-minute-walking-test; fatigability was calculated as the ratio of distance walked in the final minute to the first minute (distance walking index, DWI6−1). Fatigue was evaluated through the Modified Fatigue Impact Scale (MFIS). Spearman ρ examined the relationship among variables; linear regression analyses examined predictors of fatigue and fatigability.

**Results:**

70 pwMS (age 37.8+11years; female n=50, 71.4%, EDSS 1.5[1;2]) were enrolled. 15 (21.4%) reported significant levels of fatigue, 14 (20%) presented motor fatigability. Fatigue and motor fatigability were not significantly correlated with one another (ρ=0.100;p=0.425) or with other clinical, cognitive, radiological features. Fatigue was related to Hospital Anxiety and Depression Scale (HADS) anxiety subscale (ρ=0.375,p=0.002), Beck Depression Inventory (BDI-II) (ρ=0.543;p<0.001), neuroticism (ρ=0.313;p=0.006), and all subscales of MS-Quality-Of-Life 54. We did not find predictors of fatigability, while HADS-anxiety (b=0.76; p=0.003) and BDI-II (b=0.33; p=0.009) significantly predicted fatigue.

**Conclusions:**

Our results support different neurobiological underpinnings for motor fatigue and fatigability and reinforce the need for a multidimensional assessment from the earliest stages of the disease, to tailor therapeutic and rehabilitation strategies.

## Introduction

Multiple Sclerosis (MS) is a chronic inflammatory and neurodegenerative disease of the central nervous system [1]. Fatigue is one of the most prevalent and disabling symptoms reported by people with multiple sclerosis (pwMS), often emerging in the early stages of the disease, sometimes even before significant physical disability becomes clinically detectable [2, 3]. Fatigue is defined as a subjective sensation of exhaustion that is disproportionate to exertion, not adequately relieved by rest; it affects daily functioning, mobility, cognitive performance, social participation, and quality of life of patients [4]. It can only be measured by self-report [5] and several questionnaires have been validated to obtain clinical assessments of the level of fatigue experienced by pwMS [6, 7]. 

In contrast, motor fatigability refers to the measurable, objective decline in neuromuscular performance during sustained or repetitive motor activity [8, 9], such as the change in a performance metric during a motor-endurance task, including the 6-minute walking test [10]. Besides, it can be quantified through changes in muscle force output, electromyographic parameters, or reductions in voluntary activation assessed via techniques such as transcranial magnetic stimulation [8]. The prevalence of motor fatigability increases with physical disability and in progressive MS, but recent findings show that it is also significantly present in non-disabled pwMS [11]. 

Although the terms fatigue and motor fatigability are sometimes used interchangeably, evidence suggests that they represent partially dissociable constructs [12], with at most a moderate correlation [13]. Subjective fatigue appears to involve altered processing within central neural networks, including fronto-striatal, limbic, and thalamo-cortical systems, [14] as well as dysregulation of neurotransmitter systems such as dopamine and noradrenaline [4]. Motor fatigability, on the other hand, may reflect peripheral conduction block, impaired neuromuscular transmission, or reduced efficiency of corticomotor drive during motor tasks [5]. 

Studying the relationship between these two dimensions in early MS is clinically relevant. Differentiating subjective and objective components of fatigue may improve diagnostic precision, guide personalized rehabilitation strategies, and contribute to the identification of early biomarkers of disease progression.

Therefore, the aim of this study was to evaluate the correlations between fatigue and motor fatigability in early pwMS, and to analyse their clinical, radiological and psychological underpinnings.

## Methods

### Study design and participants

This was an observational monocentric study, performed within the Reliable study (Erapermed 2022 − 295), funded by Italian Ministry of Health, Tuscany Region. It involved patients consecutively referred to and followed-up at the MS Centre at Careggi University Hospital in Florence, Italy, who have been evaluated at IRCCS Don Gnocchi Foundation, Florence, Italy. Inclusion criteria were diagnosis of relapsing remitting MS based on the 2017 McDonald criteria, age between 18 and 65 years, Expanded Disability Status Scale (EDSS) score *≤* 2,0, disease duration *≤* 5 years and adequate mental capacity to provide informed consent. The exclusion criteria were steroid treatment within 30 days and clinical relapses within 3 months from inclusion, diagnosis of major depression, severe joint and/or bone disorders, cardiovascular diseases or other concomitant neurological diseases interfering with balance and gait, based upon clinical judgment. Participants were recruited between March 2023 and March 2025.

After receiving a full explanation of the study, each subject gave a written informed consent to participate. The study was approved by the Ethics Committee of the Tuscany Region – Central Area (CEAVC) in 2023.

## Procedures

At baseline, demographic variables, including educational and occupational history, height and weight, body mass index (BMI), lifestyle risk/protective factors and clinical data such as comorbidities and therapies were recorded. MS information, including time and type of onset, time of diagnosis, EDSS at baseline, and disease-modifying treatments (DMTs), were collected. The EDSS score [15] was determined during the clinic visit by a certified EDSS examiner. Fatigue was assessed through the Modified Fatigue Impact Scale (MFIS), and it was considered relevant when MFIS *≥* 38 [6, 16]. Cognitive function was evaluated through the Brief International Cognitive Assessment for Multiple Sclerosis (BICAMS) battery [17, 18], consisting of Symbol Digit Modalities Test (SDMT) for processing speed, the California Verbal Learning Test-2 (CVLT-2) for verbal learning/memory, and the Brief Visuospatial Memory Test-Revised (BVMT-R) for visuospatial memory. Furthermore, anxiety and depressive symptoms were assessed with the Hospital Anxiety Depression Scale (HADS) [19] and Beck Depression Inventory II (BDI-II) [20], while patients’ quality of life through the MS quality of life-54 (MSQOL-54) [21]. The Twelve-item Multiple Sclerosis Walking Scale (MSWS-12) [22] was administered to assess perceived walking ability and the subjective impact of MS on walking and related activities. A psychological assessment was conducted to evaluate personality traits using the Big Five Inventory-10 (BFI-10) [23], emotion regulation and dysregulation with the short version of the Difficulties in Emotion Regulation Scale (DERS-18) [24], psychological inflexibility and experiential avoidance with the second version of the Acceptance and Action Questionnaire (AAQ2) [25].

Gait analysis was performed at IRCCS Fondazione Don Carlo Gnocchi, Florence. Walking performance was assessed by a sensorised 6-minute walking test (6MWT). Subjects were instructed to walk at their fastest speed, and to cover as much distance as possible, according to Goldman et al., 2008 [26]. Subjects were notified, without further encouragement, about each expired minute. They walked back and forth along a 30 m hallway, turning around cones at each end, in which 9 central metres were sensorized with Optogait (Microgate) for gait assessment. The Optogait system has been validated for gait analysis and has strong validity and reliability [27]. Distances walked per minute and total distance were recorded together with all spatio-temporal parameters of all runs within the 9 m sensorized. Motor fatigability has been defined through the distance walked index (DWI) [10], calculated according to the formula (((Distance walked minute 6 - Distance walked minute 1)/Distance walked minute 1)∗100), and we defined motor fatigability as DWI 6 − 1 <-10%, as defined by Van Geel et al., 2020 [28]. 

Brain magnetic resonance imaging (MRI) scans were obtained through a Philips Ingenia 3.0 Tesla scanner. Axial T2 weighted Turbo Spin Echo, axial Fluid Attenuated Inversion Recovery (FLAIR) and high-resolution 3D sagittal T1-weighted sequence were collected following a standardized protocol of acquisition. Brain T2-hyperintense and T1-hypointense lesion volumes were quantified on FLAIR and 3D T1-weighted scans, respectively, using an automated lesion segmentation tool with manual editing for artifacts. After refilling of T1 hypointensities, normalized brain volume (NBV), grey matter volume (GMV) and white matter volume (WMV) were measured on 3D T1-weighted scans using SIENAX software [29]. 

### Statistical Analysis

Baseline characteristics were summarized using mean and standard deviation for continuous variables and frequency counts and percentages for categorical variables. Spearman ρ was calculated to examine the relationship among fatigue, motor fatigability and other clinical, radiological and psychological features. Linear regression analyses were performed, with predictors selected using backward stepwise elimination (removal criterion: *p* > 0.05) to examine predictors of fatigue and fatigability, adjusting for age, sex, EDSS, personality tracts, and walking performance on 6MWT.

All confidence intervals are two-sided and use 95% confidence levels. A two-sided p-value < 0.05 was considered statistically significant. All statistical analyses were performed with SPSS software, version 29.0 (SPSS inc.). Anonymized data, not published in the article, will be shared on reasonable request from a qualified investigator.

## Results

We enrolled 70 relapsing remitting pwMS (aged 37.8 *±* 11 years; female *n* = 50, 71.4%). The main demographic, clinical and cognitive features of the cohort are reported in Table 1, patient reported outcome scores are reported in Table 2, and main spatiotemporal parameters and total meters walked during the 6MWT in Table 3.

**Table 1:** Main demographic, clinical and cognitive features of pwMS cohort

	pwMS (*n* = 70)
Age, y, mean ± SD	37.8 *±* 11
Sex (F), n(%)	50 (71.4)
Years of schooling, y, median (IQR)	15(5)
Currently working, n(%)	61(87.1)
BMI (kg/m2), mean ± SD	23.8 *±* 4.5
EDSS, median(IQR)	1.5(0.5)
Disease duration, y, mean ± SD	2.6 *±* 1.3
SDMT, mean ± SD	60.1 *±* 10.9
DMT type (HET), n(%)	48(69.6)
No DMT	1(1.4)
Dimethyl fumarate	18(25.7)
Teriflunomide	1(1.4)
Azathioprine	1(1.4)
S1P modulators	1(1.4)
Cladribine	10(14.3)
Natalizumab	13(18.6)
Anti-CD20	25(35.7)

Abbreviations: *pwMS* person with Multiple Sclerosis, *SD* standard deviation, *IQR* interquartile range, *BMI* Body Mass Index, *EDSS* Expanded Disability Status Scale, *DMT* disease modifying treatment, *HET* highly effective treatment

**Table 2:** Patient reported outcomes results from pwMS cohort

MFIS, mean *±* SD	24.5 *±* 14.9
HADS anxiety, mean ± SD	6.3 *±* 3.8
BDI-II, mean ± SD	8.4 *±* 8.4
MSWS-12, mean ± SD	15.8 *±* 5.3

Abbreviations: *MFIS* modified fatigue impact scale, *HADS* hospital anxiety and depression scale, *BDI* beck depression inventory, *MSWS* Multiple Sclerosis Walking

**Table 3:** Main results from gait analysis during 6MWT

Total meters walked, m, mean *±* SD	605.6 *±* 70.3
Step length, cm, mean ± SD	82.1 *±* 7.6
Stride length, cm, mean ± SD	164.3 *±* 7.6
Velocity, m/s, mean ± SD	1.8 *±* 0.2
Cadence, step/min, mean ± SD	132.1 *±* 8.6
DWI6-1, mean ± SD	-5.3 *±* 6.7

Abbreviations: *6MWT* 6-minute walking test, *SD* standard deviation, *DWI6-1* distance walking index

In this cohort 15 (21.4%) pwMS reported significant levels of fatigue, as defined by MFIS *≥* 38. Moreover, 14 (20%) pwMS presented motor fatigability during 6MWT, as defined by DWI6-1*≤*-10%. Only 3 patients presented both conditions.

As showed in Table 4, we did not find any significant correlation between motor fatigue, as defined by MFIS physical subscale (MFIS-ph) score, and motor fatigability, as defined by DWI6-1 (ρ = 0.100; *p* = 0.425).

**Table 4:** Correlations of motor fatigue and fatigability measures

	DWI 6 − 1	MFIS ph	Age	T2 lesion volume	thalamic volume	6MWT meters walked	SDMT	BDI-II	HADS anxiety	BFI neurotic
DWI_6_1	1									
MFIS ph	0.100	1								
Age	-0.042	-0.120	1							
T2 lesion volume	-0.275	0.087	0.474**	1						
Thalamic volume	-0.258	-0.036	-0.284*	-0.011	1					
6MWT meters walked	0.127	**-**0.222	-0.266*	-0.037	0.177	1				
SDMT	0.105	-0.002	-0.254*	-0.023	0.447**	0.360**	1			
BDI-II	0.212	0.543**	-0.219	-0.124	-0.026	-0.186	-0.063	1		
HADS anxiety	0.167	0.375**	-0.361**	0.113	0.002	0.047	0.027	0.542**	1	
BFI neurotic	0.167	0.313**	-0.231	-0.022	-0.138	-0.040	0.038	0.331**	0.331**	1

Abbreviations: *DWI* distance walking index, *6MWT* 6-minute walking test, *MFIS* modified fatigue impact scale, *ph* physical subscale, *SDMT* symbol digit modalities test, *HADS* hospital anxiety and depression scale, *BDI* beck depression inventory, *BFI* big five inventory

*p<0.05, **p <0.001

DWI6-1 was not significantly related to any other clinical, cognitive, psychological and radiological data. Only a trend toward significance was observed for greater T2 lesion volume and motor fatigability (ρ=-0.275, *p* = 0.085).

On the other hand, MFIS-ph was significantly related with anxiety and depressive symptoms as defined by BDI-II (ρ = 0.543, *p* < 0.001), and HADS anxiety (ρ = 0.375, *p* = 0.002). It was also related to perceived walking ability, as measured by the MSWS-12 (ρ = 0.589; *p* < 0.001). Besides, motor fatigue was associated with all MSQOL-54 subscales, that is, worse pain feeling (ρ=-0.474, *p* < 0.001), impair in sexual function in both women (ρ=-0.312; *p* = 0.029) and male sex (ρ=-0.355; *p* = 0.136), less emotional well-being (ρ=-0.392; *p* < 0.001) and worse overall quality of life (ρ=-0.398; *p* < 0.001). Moreover, motor fatigue was correlated with a neurotic personality tract (ρ = 0.313; *p* = 0.013), difficulty in negative emotion regulation as defined by DERS total score (ρ=-0.436; *p* < 0.001), and all sub-items of the scale except the sub-item evaluating the lack of emotional awareness (ρ = 0.001; *p* = 0.996). Finally, greater fatigue was correlated to higher psychological inflexibility, experiential avoidance, and more potential psychological distress as defined by higher AAQ2 scores (ρ = 0.414; *p* < 0.001).

As it concerns multivariable regression analyses, we did not find any significant predictor of motor fatigability. Conversely, anxiety (b = 0.76; *p* = 0.003) and depressive symptoms (b = 0.33; *p* = 0.009) were confirmed significantly related to MFIS-ph even after correcting for age, sex, EDSS, neurotic personality tract and total meters walked in 6MWT (Table 5).

**Table 5:** Predictors of motor fatigue after backward deletion

	B	95%CI	p
HADS anxiety	0.76	0.27–1.25	**0.003**
BDI-II	0.33	0.09–0.57	**0.009**

Abbreviations: *HADS* hospital anxiety and depression scale, *BDI* beck depression inventory

## Discussion

Our results show around 20% prevalence of both motor fatigue and fatigability in a cohort of early and non-disabled pwMS, that is in line with previous literature [3, 10]. 

We did not find significant correlation between these two conditions and only 3 pwMS presented both. Previous evidence supports a complex and non-linear relationship between subjective fatigue and motor fatigability in pwMS, with some individuals demonstrating high levels of perceived fatigue while displaying preserved motor endurance, whereas others exhibiting marked motor fatigability despite reporting minimal subjective fatigue [30]. Our findings could suggest that, in early, non-disabled pwMS these two conditions are particularly disconnected, presumably due to the involvement of distinct neurophysiological mechanisms.

We did not find any significant predictors or correlations of motor fatigability with the other clinical, cognitive, psychological, or radiological assessments. This could be related to the objective nature of motor fatigability and to the absence of overt disability in our cohort, which represents an early disease stage in which substantial lesion burden and physical deconditioning may not yet have developed. Nevertheless, we observed a trend toward a greater T2 lesion load being associated with increased motor fatigability. This supports the idea that motor fatigability has an objective basis, being more directly linked to structural and functional alterations in neural systems involved in neuromuscular control, leading to conduction slowing, reduced axonal integrity, and impaired corticospinal recruitment during sustained motor output [12]. 

Conversely, motor fatigue was significantly related to anxiety and depressive symptoms, neurotic personality, difficulty in emotion regulation and psychological inflexibility. However, after correcting for age, sex, EDSS score and performance in 6MWT, only anxiety and depressive symptoms still predicted motor fatigue. These results align with the recently postulated theories of fatigue as failure in interoceptive insight, that is, the metacognitive aspect of interoception. Specifically, fatigue would reflect the metacognitive diagnosis that the brain is failing to exert control over bodily states and lacks any action at its disposal to overcome a state of dyshomeostasis [31]. Many potential mechanisms have been hypothesized and variously proven to be linked to fatigue. White and grey matter lesions, and even more microstructural changes in the normal appearing white matter, but also peripheral and central nervous system inflammation, would cooperate to determine the loss of key network function [31]. Particularly, the alteration of monoaminergic neurotransmission systems involved in motivation (dopamine), arousal (norepinephrine) and mood (serotonin) in key regions such as the prefrontal cortex, anterior cingulate cortex and anterior insula, hypothalamus and brainstem, would determine the failure of interoceptive insight and indeed the perception of fatigue [32]. 

Notably, our results show that since the earliest phase of the disease, pwMS can present motor fatigue and/or fatigability, even in absence of overt disability.

Besides, we confirm previous findings showing that fatigue, anxiety and depression are closely interrelated and commonly experienced in the early months following MS diagnosis [33] and that depressive and anxiety symptoms are not associated with disability level, but rather with fatigue, particularly in younger patients [34]. The tendency for symptoms to co-occur, influencing one another, and acting both as causes and as effects, may produce additive or amplified negative impacts [35], raising the need for optimizing symptom management with therapeutic approaches taking into account these complex interactions and ideally allowing for their simultaneous treatment [36]. 

Moreover, we found no correlation between cognitive performance and motor fatigue or fatigability. This result may reflect the early stage of the disease and the absence of overt cognitive or motor impairment in our cohort. Conversely, we observed a relationship between cognitive performance, as measured by the SDMT, and walking performance during the 6MWT. The link between gait and cognition is now well established [37] and is highly relevant in the daily life of pwMS [38]. Identifying this association at such an early stage, in patients without evident motor or cognitive deficits, is particularly noteworthy and warrants further investigation.

Collectively, these findings suggest that pwMS should be assessed as comprehensively as possible, allowing clinicians to tailor preventive, therapeutic, and rehabilitative strategies to individual needs. In this context, the development of digital tools could enable a broader and more granular assessment of patients in their daily lives, potentially facilitating the detection of silent progression, and highlighting the potential prognostic relevance of specific features, such as motor fatigability, even in the earliest stages of the disease [39]. Furthermore, prehabilitation strategies could help address subtle motor and cognitive dysfunction, as well as hidden symptoms that affect patients’ everyday functioning [40]. 

Our study has several limitations. First, the relatively small cohort size and the cross-sectional design, that could limit the possibility to draw strong conclusions and to identify clear predictors, especially for motor fatigability. Therefore, the results should be considered exploratory and should be confirmed in larger cohorts. Another limitation is the use of the MFIS-physical subscale as a measure of motor fatigue, in the absence of a validated threshold. Moreover, DWI6-1 could be a less sensitive measure for motor fatigability compared to other ways of assessing fatigability such as superficial electromyography, or the analysis of kinematics of gait to evaluate changes in quality of gait throughout the endurance exercise, thus leading to a potential underestimation of the real prevalence of motor fatigability in the cohort. Regarding MRI assessment, some limitations should be noted: the absence of spinal cord assessment and the lack of certain advanced metrics, such as evaluation of cortical lesions or paramagnetic rim lesions.

Despite the above limitations, our study adds to the previous literature exploring in detail a wide range of clinical, psychological, cognitive, and MRI features for their potential association with motor fatigue and fatigability. Besides, by focusing on early, non-disabled pwMS, this multidimensional approach could represent an instrument for screening individuals who are most likely to benefit from prehabilitation strategies, and for tailoring subsequent treatment strategies.

## Conclusion

In conclusion, we found that in early, non-disabled pwMS, motor fatigue and fatigability were significantly prevalent, but they tend not to occur in the same individuals. We did not find predictors of fatigability, while anxiety and depressive symptoms strongly predicted motor fatigue. These results have practical implications in patient management, highlighting the need for a multidimensional assessment of pwMS since the earliest stage of the disease, in order to define a personalized therapeutic and rehabilitation approach.

## Declarations

### Data sharing

Data will be available under reasonable demand to the corresponding author.
